# Time Trends and Patterns of Reported Egg Consumption in the U.S. by Sociodemographic Characteristics

**DOI:** 10.3390/nu9040333

**Published:** 2017-03-28

**Authors:** Zach Conrad, LuAnn K. Johnson, James N. Roemmich, WenYen Juan, Lisa Jahns

**Affiliations:** 1 Grand Forks Human Nutrition Research Center, US Department of Agriculture, Agricultural Research Service, 2420 2nd Avenue N, Grand Forks, ND 58203-9034, USA; luann.johnson@ars.usda.gov (L.K.J.); james.roemmich@ars.usda.gov (J.N.R.); lisa.jahns@ars.usda.gov (L.J.); 2 Center for Food Safety and Applied Nutrition, US Department of Health and Human Services, Food and Drug Administration 5001 Campus Drive, College Park, MD 20740, USA; wenyen.juan@fda.hhs.gov

**Keywords:** eggs, trends, sociodemographic characteristics, dietary intake

## Abstract

Eggs have the potential to contribute essential nutrients to nutritionally vulnerable populations on limited food budgets. Further research is needed to better understand patterns of egg consumption across diverse sociodemographic groups in order to inform clinical practice to improve nutrient adequacy. Data on demographics and egg intake of 29,694 U.S. adults were obtained from the National Health and Nutrition Examination Survey, 2001–2012. The National Cancer Institute’s usual intake methodology was used to estimate the distribution of egg intake. Linear and logistic regression models were used to test for time trends in egg consumption and for differences between sociodemographic groups. The proportion of the U.S. population, overall (21%–22%; *p* = 0.311) and by sociodemographic group (*p* > 0.05 for all groups), that reported consuming eggs remained unchanged from 2001 to 2012. Mean egg consumption increased overall from 23.0 (95% CI, 20.8–25.2) g/day in 2001–2002 to 25.5 (22.7–28.4) g/day in 2011–2012 (*p* = 0.012), but not among food insecure individuals (*p* = 0.816) and Supplemental Nutrition Assistance Program (SNAP) participants (*p* = 0.399). No differences in the odds of egg consumption were observed by income level, food security status, or SNAP participation status (*p* > 0.05 for all groups). Given the nutritional benefits of eggs, as well as their low cost and culinary versatility, the results presented here have important implications for reducing disparities in health outcomes and diet quality, in particular among food insecure individuals and SNAP participants. Further research is needed to examine factors that influence egg consumption and associated nutrient intake, and to identify potential barriers to increasing egg consumption, such as egg price changes, across diverse sociodemographic groups.

## 1. Introduction

Eggs are low-cost and rich in many important nutrients, including all essential amino acids [1,2], choline, B vitamins, vitamin A, iron, vitamin D [3], and the xanthophyll carotenoids lutein and zeaxanthin [4,5,6]. Evidence also shows that consuming eggs increases the bioavailability of co-consumed carotenoids [7] and vitamin E [8], and also contributes to satiety, an important factor for weight control [9]. 

The most recently available data (2001–2008) on the prevalence of egg consumption show that approximately 20% of the U.S. population reported consuming eggs on a given day [10], which is unchanged from earlier (1988–1994) estimates [11]. On a weight basis, Rehm et al. reported a positive trend in egg consumption from 1999–2000 to 2011–2012, albeit of small magnitude [12].

In the U.S., food and nutrient intakes vary by sociodemographic characteristics such as age, income, education level, and race-ethnicity [13,14]. For instance, Mexican–American and non-Hispanic black children are most likely to have serum vitamin D concentrations levels below the at-risk cutoff [13]. Individuals from low-income and food insecure households, even those participating in federal food assistance programs, have lower quality diets compared to the general population [15]. 

Data from 2003 to 2006 show that eggs contribute important nutrients to minority race-ethnic groups [16,17] and overweight and obese women [17]. Others demonstrated that egg consumption by Supplemental Nutrition Assistance Program (SNAP) participants did not differ from income-eligible non-SNAP participants [18].

Given that eggs are nutritious, low cost, and a part of a wide range of cultural food menus, they have the potential to contribute valuable nutrients to diverse populations including nutritionally vulnerable groups with limited food budgets. Despite recent analyses of population-level trends in egg consumption, further research is needed to better understand patterns of egg consumption across diverse sociodemographic groups, including nutritionally vulnerable groups, in order to inform clinical practice to improve nutrient adequacy. The goal of this research was to (1) describe time trends in frequency and amount of egg consumption; and (2) describe differences in egg consumption by sex, age, income, education, race-ethnicity, SNAP participation status, and food security status. We expected to observe a moderate increase in overall egg consumption over time and heterogeneous trends across sociodemographic groups.

## 2. Materials and Methods

### 2.1. Survey Design

Data were derived from the National Health and Nutrition Examination Survey (NHANES) and its dietary survey component, the What We Eat In America (WWEIA) survey. NHANES uses a complex, multistage sampling design and is representative of the civilian non-institutionalized U.S. population. Sociodemographic groups of public health interest are oversampled. Data are collected continuously and released in two-year cycles. The NHANES survey includes a detailed medical evaluation, demographic and health and behavior questionnaires, physical activity assessment, and dietary recalls collected by WWEIA. Details may be found elsewhere [19]. The present analyses used data from adults aged 20+ in the 2001–2002, 2003–2004, 2005–2006, 2007–2008, 2009–2010, and 2011–2012 data releases. NHANES protocols were approved by the National Center for Health Statistics Ethics Review Board and all participants provided written consent. 

### 2.2. Egg Consumption

In 2001–2002 a single day’s 24-h dietary recall was collected. WWEIA 2003–2012 consists of two non-consecutive, interviewer-administered 24-h dietary recalls using the U.S. Department of Agriculture (USDA) Automated Multiple-Pass Method [20]. The first interview is administered in-person and the second by telephone 3–10 days later. Only the data collected from the first 24-h recall was used to estimate intake; data from the second 24-h recall was used to estimate the within-person variation in intake [21]. In 2003–2004 and 2005–2006, a food propensity questionnaire was administered [22]. One of the questions asked was “Over the past 12 months… How often did you eat eggs, egg whites, or egg substitutes (NOT counting eggs in baked goods and desserts)? (Please include eggs in salads, quiche, and souffles)”. Nearly all (94%) individuals reported eating eggs at some point in the previous year, so per capita estimates are presented. The USDA Food and Nutrient Database for Dietary Studies (FNDDS) [23] was used to identify eggs and egg mixtures that were composed primarily of eggs (i.e., primarily classified as either “eggs” or “egg mixtures” by FNDDS staff using standardized procedures; specific FNDDS food codes included in this analysis are listed below), such as cheese omelets and egg salad sandwiches. Dishes made with egg whites were included, and egg substitutes were excluded because of their distinct nutrient profile and extremely small consumption frequency and amount (less than 0.5% of the sample reported consuming egg substitutes on the survey day, with a mean per capita intake of <0.5 grams/day). FNDDS food codes included in this analysis were 31101010–31111020 and 32101500–32401000, and these represented 178 discrete food items reported consumed in WWEIA 2001–2012.

### 2.3. Sociodemographic Characteristics

Household income was categorized using the ratio of family income to the federal poverty threshold, known as the income-to-poverty ratio (IPR) [24]. Income categories were IPR 0–1.30, 1.31–1.85, and >1.85, with the lowest category representing individuals who live in households that satisfy the income requirement for participating in the Supplemental Nutrition Assistance Program (SNAP), a federal food assistance program [25]. Education was categorized as <high school diploma, high school diploma or equivalent, or some post-secondary education. SNAP participation was defined as an individual currently receiving benefits. Individuals with a IPR ≤ 1.3 but reporting that they were not authorized to receive food stamps or had not received food assistance during the last 31 days were categorized as “income-eligible non-participants”. Food security status was assessed in all waves using the 10-item U.S. Adult Food Security Survey Module [26] and categorized into fully food secure, marginally food secure, or low food security status. 

NHANES is designed to oversample some sociodemographic groups in order to increase reliability and precision. Mexican-Americans were oversampled from 2001 to 2006, and this group was expanded to include all Hispanic persons beginning in 2007–2008 [27]. The National Center for Health Statistics provides guidance for researchers when analyzing multiple NHANES waves, and recommends that, when waves 2005–2006 and 2007–2008 are analyzed in the same study, the Mexican-American category be used instead of the Hispanics category due to the different sampling method for Hispanic persons between these waves [27]. Further changes to the NHANES sampling design were implemented in 2011–2012 that included oversampling Asians [28]. Therefore, in accordance with recommended analytical procedures, when reporting consumption by race-ethnicity we removed from analysis all individuals (*n* = 3909) who reported any category other than non-Hispanic black, Mexican–American, or non-Hispanic white. The final sample size used in this analysis was *n* = 29,694.

### 2.4. Statistical Analysis

The percentage of individuals who reported eating eggs and per capita consumption amounts were age-adjusted using the method and weights provided by Klein and Schoenborn [29]. The USDA standardizes the weight of eggs from disparate food sources (e.g., quiche, scrambled eggs, whole eggs) as one ounce-equivalent equals one egg which equals approximately 50 grams of eggs [30]; in order to provide relevance to an international audience we report consumption amounts in grams. Linear regression models with weights equal to the standard error of each estimate were used to test for linear trends across survey cycles for each sociodemographic group. Logistic regression was used to test whether the odds of consuming eggs differed between the levels of age, sex, race-ethnicity, income, education, food assistance participation, and food security. Age, sex, race-ethnicity, and survey year were included as covariates in all models.

The distribution of egg intake for each sociodemographic group was estimated using the National Cancer Institute’s (NCI) usual intake methodology [31]. The NCI method uses information from two 24-h recalls to estimate the within-individual variability in intake and requires two Statistical Analysis System (SAS) macros: MIXTRAN and DISTRIB. Because eggs are not usually consumed daily, MIXTRAN was used to fit a two-part model, in which the probability of consuming eggs was allowed to be correlated with the amount consumed [31]. Covariates were age, sex, race-ethnicity, survey cycle, and whether the intake day was a weekday (Monday–Thursday) or weekend (Friday–Sunday). Standard errors were estimated using the balanced repeated replication method.

All statistical analyses were completed using SAS V9.4 (SAS Institute, Inc., Cary, NC, USA), and day 1 sample weights were used to account for differential probabilities of selection, nonresponse, and noncoverage.

## 3. Results

### 3.1. Time Trends in Egg Consumption by Sociodemographic Group

#### 3.1.1. Trends in the Percentage of Individuals Reporting Consuming Eggs on a Given Day

The percentage of the U.S. population reporting egg consumption on day 1 of the survey did not change from 2001–2002 to 2011–2012, overall (21%–22%; *p* = 0.311) or by any sociodemographic subgroup (Table S1). 

#### 3.1.2. Trends in the Amount of Eggs Consumed Per Day

**Figure 1:**
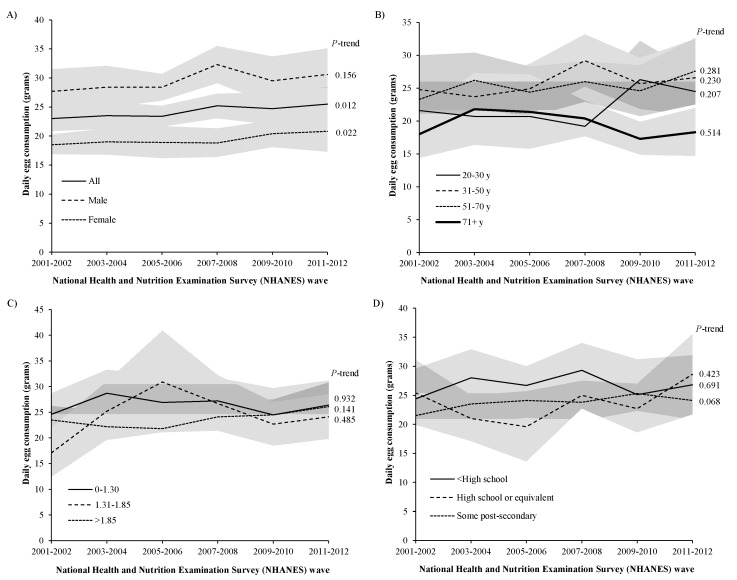

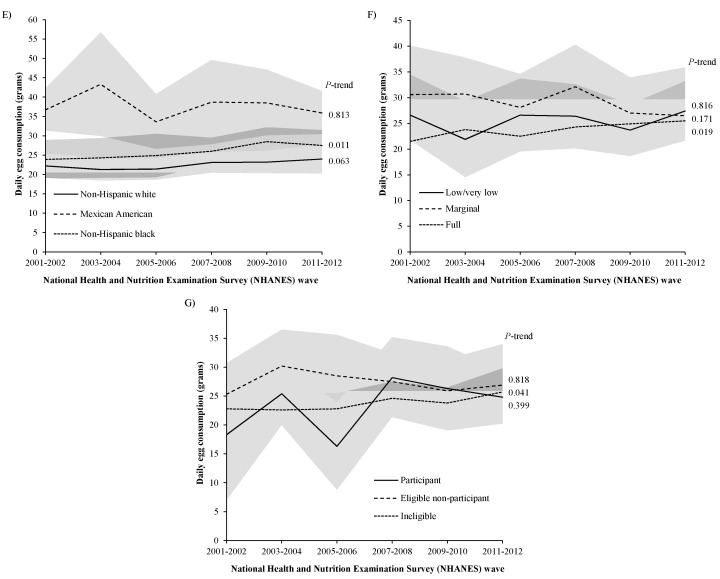
Mean age-adjusted amount (grams) of eggs consumed per capita by (**A**) sex; (**B**) age group; (**C**) income-to-poverty-ratio; (**D**) education; (**E**) race-ethnicity; (**F**) food security; and (**G**) Supplemental Nutrition Assistance Program (SNAP) participation, National Health and Nutrition Examination Survey (NHANES) 2001–2012 (*n* = 29,694).

Overall per capita egg consumption increased by 11% from 2001–2002 to 2011–2012 (*p* = 0.012), and egg consumption increased among women (*p* = 0.022) but not men (*p* = 0.156) (Figure 1A). No significant time trends were observed by age group (Figure 1B), income level (Figure 1C), and education (Figure 1D). Non-Hispanic black individuals increased consumption by 15% (*p* = 0.011), but there was no significant time trend among Mexican-Americans or non-Hispanic whites (Figure 1E). Individuals classified as fully food secure reported a 20% increase (*p* = 0.019) in egg consumption, but no time trends were observed among individuals of marginal or low/very low food security status (Figure 1F). Individuals who were income-ineligible for participation in SNAP increased their egg consumption from 2001–2002 to 2011–2012 (*p* = 0.041), but no time trends were observed for SNAP participants and SNAP-eligible non-participants (Figure 1G). No groups decreased consumption of eggs. 

### 3.2. Patterns of Reported Egg Consumption by Sociodemographic Group

#### 3.2.1. Odds of Consuming Eggs

Women had lower odds of consuming any eggs on the first day of dietary recall compared to men (OR 0.82, 95% CI 0.76–0.88). Compared to individuals 20–30 years old, those who were 51–70 years old had the greatest odds of consuming eggs (1.66, 1.48–1.86), followed by those 71+ (1.58, 1.4–1.79), and those 31–50 years old (1.37, 1.23–1.52) (Table 1). Compared to non-Hispanic whites, the odds of egg consumption were 1.79 (1.55–2.05) fold greater among Mexican-Americans and 1.33 (1.20–1.46) fold greater among non-Hispanic blacks. No differences in the odds of egg consumption were observed by income, education, food security status, or SNAP participation status.

#### 3.2.2. Mean and Percentiles of Daily Egg Consumption

Overall, individuals consumed 24 ± 0.9 g eggs/day (one egg equals ~50 g [32]; Table 2). Women consumed 10 g/day less eggs than men (*p* < 0.001), and those with less than high school education consumed 4 g/day more than those with postsecondary education (*p* = 0.02). Mexican-Americans consumed a mean of 37.0 ± 1.9 g/day, 14 g/day more than non-Hispanic whites (*p* < 0.001), and non-Hispanic blacks consumed 27.5 ± 1.0 g/day of eggs/day, 4.5 g/day more than non-Hispanic whites (*p* = 0.005). There was no difference in the amount of eggs consumed by age, income, food security status, or SNAP participation status. 

## 4. Discussion

In this cross-sectional, nationally representative study of nearly 30,000 U.S. adults that provided dietary data from 2001–2002 to 2011–2012, approximately one-fifth of individuals reported consuming eggs on the day of the survey in each survey wave. Over this twelve year period, mean per capita egg consumption increased overall and among select sociodemographic groups, including fully food secure individuals and SNAP-ineligible individuals; yet no difference in mean daily intake was observed between levels of food security and SNAP participation status. These findings represent the most up-to-date trends and patterns of egg consumption across diverse sociodemographic groups in the U.S. 

Consistent with these findings, Nicklas et al. reported that 20% of adults in NHANES 2001–2008 reported consuming eggs on the day of the survey [10], and Rehm et al. reported a positive trend in overall egg consumption using NHANES data from 1999–2000 to 2011–2012 [12]. Cifelli et al. estimated daily egg consumption of 0.52 servings/day (equivalent to 26 g/day) from 2007 to 2010 (NHANES) [33], which is similar to Rhem et al. (0.50–0.51 servings/day from 2007–2012, equivalent to 25–25.5 g/day) [12] and to the present study (24.7–25.5 g/day from 2001 to 2012). Consistent with others [18], we observed no difference in mean daily egg consumption between SNAP participants and eligible non-SNAP participants.

The current work extends this area of research by being the first study to evaluate trends and absolute consumption of eggs across a wide range of sociodemographic groups and doing so over a twelve year contemporary period. Eggs are among the highest quality protein sources, are one of the best sources of micronutrients per calorie, and increase the bioavailability of some co-consumed nutrients (carotenoids [7] and vitamin E [8]). Egg consumption promotes positive health outcomes at all ages and all stages of life [34], and lowers the risk of some cardiovascular events [35]. Given these health benefits, the results presented here have important implications for reducing disparities in health outcomes and diet quality, in particular among food insecure individuals and SNAP participants who did not increase their consumption of eggs from 2001–2012, whereas their fully food secure and SNAP-ineligible counterparts did. On the one hand, given that chronic disease risk is greater and diet quality is lower among individuals in lower income strata [36,37], it is concerning that these individuals are forgoing the additional nutritional benefits that could otherwise be provided by consuming more eggs; yet on the other hand, these results show that more can be done to increase egg consumption among these nutritionally vulnerable groups. Indeed, given their low cost and culinary versatility [34], eggs may be an ideal food for low-income consumers on fixed budgets. 

A potential barrier to increasing the consumption of eggs among low-income individuals is related to the volatility of egg prices, which is more severe than all other major food groups [38,39]. For example, the mean annual change (upward or downward) in retail price from 1995–2015 was 2.5% for all foods but 5% for eggs specifically [39]. Although the own-price elasticity of eggs (the change in consumption of eggs related to the change in their price) is lower than other foods (0.27% change in consumption for every 1% change in price) [40], dramatic price changes can still elicit meaningful changes in consumption. Much of the annual price volatility of eggs can be explained by the negative impact that major disease outbreaks have had on poultry flocks, most recently in 2003–2004, 2007–2008, and 2014–2015, the most recent of which caused the loss of about 33 million (11%) egg laying hens in the U.S. [38]; as a result, the price of eggs increased by 14%, 29%, and 8.4% in 2003, 2007, and 2014, respectively [39]. Further research is needed to quantify the socioeconomic differences in the own-price elasticity of eggs. Additionally, more research is needed to examine the heterogeneity of the *cross*-price elasticity of eggs (the change in the consumption of eggs related to the change in price of other foods) across sociodemographic groups, in order to better understand how the price of other foods affects egg consumption across different consumer groups. Additional barriers to increasing egg consumption among low-income individuals may be the neighborhood proximity to food retail outlets, availability of transportation, time and convenience of food preparation, and access to cooking supplies and appliances.

For the general population, barriers to increasing the consumption of eggs include their historical association with cholesterol and thus cardiovascular disease risk [41]. While eggs do contain a substantial amount of cholesterol, a consistent body of research now shows little evidence to support a clinically significant relationship between dietary cholesterol and serum cholesterol [42]. Rather than limit dietary cholesterol to a specified threshold, the most recent iteration of the Dietary Guidelines for Americans advises consumers to replace their intake of saturated fatty acids with unsaturated fatty acids (especially polyunsaturated fatty acids) in order to reduce circulating total and low-density lipoprotein (LDL)-cholesterol [43]. Still, sustained marketing efforts may be needed to fully overcome the negative public association between dietary cholesterol (and eggs) and cardiovascular disease. 

Continued advancements in nutrient-enriched eggs (for example, eggs high in α-linolenic acid and docosahexaenoic acid [44], folate [45], iodine [46], and some lipophilic antioxidants [47]) may appeal to some consumer segments. Yet the potential market for these specialty products is not well understood, and their production partly depends on the availability of specific poultry feed formulations and supply chain infrastructure, and is complicated by the recent downsizing of poultry flocks noted above.

Despite the differences we observed in egg consumption across several sociodemographic groups, some of these differences were of small magnitude (up to 14 grams/day) and the effects on health outcomes may be limited. Previous studies have shown no appreciable effect on risk of type II diabetes [48], coronary heart disease, and stroke [49] at consumption amounts of less than one-half egg (~25 grams) or one egg (~50 grams) per day. However, little is known about how small differences in egg consumption affect the probability of nutrient inadequacy, particularly among nutritionally vulnerable populations, which calls for further investigation.

This study does have limitations that warrant discussion. Participants in surveys may not accurately report their food intake in order to simplify the survey process or to impress the interviewer [50,51], and some have specifically called into question the validity of self-reported energy intake [52]. However, self-reported dietary data are nonetheless useful in characterizing food intake patterns [53]. Additionally, the focus of this study was exclusively on shell egg dishes (such as omelets and quiche, for which eggs were the main ingredient) rather than eggs in mixed dishes (such as baked goods, for which eggs are a minor ingredient), so not all egg consumption was measured. Because eggs are used as an ingredient in a wide variety of dishes that can be categorized as different food groups with disparate nutrient profiles, we limited this analysis to shell eggs in order to improve interpretability and maintain relevance to dietary recommendations. In addition, these findings are only reflective of the U.S. population; egg consumption in other countries may differ.

To the best of our knowledge, this is the first study to examine contemporary trends and patterns of egg consumption by sociodemographic group. The nationally representative design of the dietary survey and large sample size (nearly 30,000 adults) make these findings generalizable to the U.S. adult population. Egg consumption was computed using nearly 180 discrete food items for which eggs was the dominant ingredient, making these findings relevant to a wide variety of consumer food purchases.

## 5. Conclusions

The proportion of U.S. adults that reported consuming eggs on any given day remained unchanged from 2001–2002 to 2011–2012. However, mean egg consumption increased over this twelve year period, overall and among select sociodemographic groups, but not among nutritionally vulnerable food insecure individuals and SNAP participants. Given the nutritional benefits of eggs, as well as their low cost and culinary versatility, the results presented here have important implications for reducing disparities in health outcomes and diet quality, in particular among food insecure individuals and SNAP participants. Further research is needed to examine factors that influence egg consumption and associated nutrient intake. Additionally, more information is needed to identify barriers of egg consumption, and targeted and sustained marketing efforts are needed to overcome these barriers.

## Figures and Tables

**Table 1:** Odds of egg consumption by sociodemographic group, NHANES 2001–2012 (*n* = 29,694).

Sociodemographic Group	OR (95% CI)
Sex	
Men	1.00
Women	0.82 (0.76–0.88)
Age (years)	
20–30	1.00
31–50	1.37 (1.23–1.52)
51–70	1.66 (1.48–1.86)
71+	1.58 (1.40–1.79)
Income-to-poverty ratio	
>1.85	1.00
1.31–1.85	1.09 (0.97–1.23)
0–1.30	1.04 (0.95–1.14)
Education *	
Post-secondary	1.00
High school or equivalent	0.98 (0.88–1.08)
<High school	1.03 (0.93–1.13)
Race-ethnicity	
Non-Hispanic white	1.00
Mexican-American	1.79 (1.55–2.05)
Non-Hispanic black	1.33 (1.20–1.46)
Food Security *	
Full	1.00
Marginal	1.08 (0.94–1.24)
Low/very low	0.94 (0.84–1.06)
SNAP participation *	
Participant	1.00
Eligible non-participant	1.06 (0.95–1.17)
Ineligible	0.97 (0.85–1.10)

* Adjusted for sex, age, and race-ethnicity.

**Table 2:** Mean and percentiles of egg consumption by sociodemographic group, NHANES 2001–2012 (*n* = 29,694).

	Mean	Median	10th Percentile	90th Percentile	*p*—Mean Difference *
	Grams/Day ± Standard Error				
All	24.1 ± 0.9	17.1 ± 0.8	4.3 ± 0.8	53.3 ± 4.2	
Sex					
Men	29.0 ± 1.1	21.4 ± 0.9	5.6 ± 1.0	62.8 ± 5.0	
Women	19.6 ± 0.8	13.9 ± 0.7	3.6 ± 0.7	43.2 ± 3.6	<0.01
Age (years)					
20–30	22.4 ± 1.1	15.2 ± 1.0	3.7 ± 0.7	50.6 ± 4.3	
31–50	25.2 ± 1.2	17.9 ± 0.9	4.5 ± 0.8	55.5 ± 4.8	0.26
51–70	25.2 ± 1.1	18.4 ± 0.8	4.8 ± 0.9	54.7 ± 4.4	0.22
71+	20.2 ± 0.9	14.5 ± 0.8	3.7 ± 0.7	44.2 ± 3.4	0.36
Income-to-poverty ratio					
>1.85	23.7 ± 0.9	16.8 ± 0.8	4.3 ± 0.8	52.2 ± 3.9	
1.31–1.85	24.5 ± 1.2	17.5 ± 1.0	4.4 ± 0.8	54.0 ± 3.9	1.00
0–1.30	25.4 ± 1.0	18.0 ± 1.1	4.5 ± 0.8	56.3 ± 3.8	0.41
Education					
Post-secondary	23.2 ± 1.0	16.5 ± 0.8	4.2 ± 0.8	51.4 ± 4.3	
High school or equivalent	23.9 ± 1.2	16.9 ± 1.1	4.2 ± 0.8	53.0 ± 4.3	1.00
<High school	27.2 ± 1.2	19.8 ± 1.0	5.0 ± 0.9	59.4 ± 4.5	0.02
Race-ethnicity					
Non-Hispanic white	23.0 ± 0.9	15.6 ± 0.9	3.4 ± 0.8	52.8 ± 3.9	
Mexican-American	37.0 ± 1.9	28.1 ± 1.7	6.8 ± 1.4	79.6 ± 5.9	<0.01
Non-Hispanic black	27.5 ± 1.0	19.8 ± 1.1	4.5 ± 1.1	61.2 ± 3.9	<0.01
Food Security					
Full	23.6 ± 0.9	16.8 ± 0.8	4.3 ± 0.8	52.0 ± 4.1	
Marginal	27.1 ± 1.6	19.5 ± 1.4	4.9 ± 1.0	59.7 ± 5.1	0.11
Low/very low	26.0 ± 1.3	18.4 ± 1.2	4.7 ± 1.0	57.2 ± 4.7	0.26
SNAP participation					
Ineligible	23.8 ± 0.9	16.9 ± 0.8	4.3 ± 0.8	52.6 ± 4.0	
Eligible non-participant	25.2 ± 1.1	17.8 ± 1.0	4.4 ± 0.8	56.0 ± 4.2	0.65
Participant	25.5 ± 1.5	18.0 ± 1.5	4.5 ± 0.9	56.4 ± 4.7	0.66

Adjusted for sex, age, race-ethnicity, and survey wave. Note that 50 grams of eggs equals approximately one egg or one ounce-equivalent of eggs. * Compared to reference group.

## Supplementary Materials

http://www.mdpi.com/2072-6643/9/4/333/s1; Table S1: Percentage of individuals reporting egg consumption on day 1 by sociodemographic group, NHANES 2001–2012 (*n* = 29,694).

## Abbreviations

The following abbreviations are used in this manuscript:

FNDDS	Food and Nutrient Database for Dietary Studies
NCI	National Cancer Institute
NHANES	National Health and Nutrition Examination Survey
SNAP	Supplemental Nutrition Assistance Program
WWEIA	What We Eat In America
