# Supplementary material for: Time Trends and Patterns of Reported Egg Consumption in the U.S. by Sociodemographic Characteristics
Source: Nutrients. 2017 Mar 28;9(4):333. doi: 10.3390/nu9040333 (PMC5409672; doi:10.3390/nu9040333)
Supplement: Supplementary file 1 [file nutrients-09-00333-s001.docx]

Supplementary Materials: Time Trends and Patterns of Reported Egg Consumption in the U.S. by Sociodemographic Characteristics

Zach Conrad, LuAnn K. Johnson, James N. Roemmich, WenYen Juan and Lisa Jahns

**Table S1.** Percentage of individuals reporting egg consumption on day 1 by sociodemographic group, NHANES 2001–2012 (*n* = 29,694).

|  | **2001–2002** | **2003–2004** | **2005–2006** | **2007–2008** | **2009–2010** | **2011–2012** | ***p*-trend** |
| --- | --- | --- | --- | --- | --- | --- | --- |
|  | % (95% CI) | | | | | |  |
| All | 20.8 (18.8-22.8) | 20.9 (19.1–22.7) | 20.5 (18.9–22.0) | 21.2 (19.2–23.1) | 20.5 (18.0–22.9) | 21.6 (19.8–23.4) | 0.311 |
| Gender |  |  |  |  |  |  |  |
| Men | 21.8 (18.8–24.9) | 22.2 (19.6–24.8) | 22.2 (20.6–23.7) | 24.1 (21.6–26.5) | 22.4 (19.5–25.2) | 23.3 (21.0–25.5) | 0.194 |
| Women | 19.8 (18.2–21.5) | 19.7 (17.4–22.1) | 19.0 (16.6–21.4) | 18.6 (16.4–20.9) | 18.8 (15.9–21.7) | 20.1 (17.3–22.8) | 0.622 |
| Age |  |  |  |  |  |  |  |
| 20–30 | 17.4 (14.9–20.0) | 15.6 (11.9–19.3) | 16.1 (13.0–19.1) | 14.4 (11.8–16.9) | 19.2 (15.4–23.1) | 17.7 (15.8–19.6) | 0.588 |
| 31–50 | 21.8 (18.5–25.1) | 19.5 (17.3–21.6) | 20.1 (17.2–23.0) | 23.0 (20.1–25.8) | 19.6 (16.8–22.3) | 21.9 (19.1–24.7) | 0.710 |
| 51–70 | 21.6 (17.7–25.6) | 26.0 (22.0–30.1) | 23.2 (20.5–26.0) | 23.2 (20.8–25.6) | 23.0 (19.3–26.6) | 24.4 (20.6–28.2) | 0.747 |
| 71+ | 21.7 (16.7–26.8) | 23.7 (19.7–27.7) | 23.7 (17.9–29.6) | 22.9 (19.3–26.6) | 19.5 (16.8–22.2 ) | 22.0 (18.0–26.0) | 0.329 |
| Income |  |  |  |  |  |  |  |
| >1.85% poverty | 20.8 (18.1–23.4) | 20.3 (18.1–22.6) | 19.7 (18.0–21.5) | 20.2 (17.5–23.0) | 19.6 (17.0–22.2) | 21.7 (19.1–24.4) | 0.231 |
| 1.31%–1.85% poverty | 17.9 (13.8–22.1) | 23.3 (19.0–27.7) | 24.5 (17.5–31.6) | 25.8 (22.3–29.2) | 20.3 (18.1–22.6) | 24.0 (20.0–28.1) | 0.411 |
| 0–1.30% poverty | 22.1 (19.3–24-8) | 22.9 (20.1–25.6) | 21.9 (17.8–26.0) | 22.7 (19.5–25.9) | 21.5 (17.2–25.7) | 21.5 (19.0–24.0) | 0.696 |
| Education |  |  |  |  |  |  |  |
| <High school | 23.2 (19.4–27.0) | 23.3 (19.8–26.9) | 22.4 (19.7–25.2) | 25.7 (22.0–29.4) | 21.4 (17.3–25.5) | 23.2 (20.2–26.1) | 0.904 |
| High school or equivalent | 23.4 (20.7–26.2) | 18.8 (16.3–21.3) | 16.2 (12.0–20.4) | 22.1 (19.7–24.4) | 18.8 (15.6–22.1) | 22.5 (19.3–25.8) | 0.997 |
| Post-secondary | 18.9 (16.4–21.4) | 21.3 (18.5–24.1) | 21.7 (19.1–24.3) | 19.1 (16.7–21.6) | 20.8 (18.3–23.2) | 20.8 (18.6–23.0) | 0.598 |
| Race-ethnicity |  |  |  |  |  |  |  |
| Non-Hispanic white | 19.3 (16.5–22.0) | 19.0 (16.7–21.4) | 19.0 (16.6–21.4) | 19.6 (16.9–22.4) | 18.7 (16.3–21.1) | 20.5 (18.0–22.9) | 0.337 |
| Mexican American | 35.0 (29.7–40.2) | 33.5 (25.5–41.4) | 27.5 (22.3–32.7) | 30.1 (24.6–35.6) | 29.2 (23.0–35.3) | 28.7 (24.2–33.1) | 0.088 |
| Non-Hispanic black | 24.4 (20.9–27.9) | 23.5 (19.6–27.4) | 23.3 (19.6–27.0) | 23.8 (21.5–26.2) | 25.4 (21.3–29.5) | 23.7 (22.0–25.3) | 0.990 |
| Food Security |  |  |  |  |  |  |  |
| Full | 19.9 (17.6–22.3) | 21.1 (18.9–23.2) | 19.8 (18.1–21.5) | 20.8 (18.5–23.1) | 20.5 (18.0–23.0) | 21.7 (19.2–24.2) | 0.226 |
| Marginal | 24.5 (19.4–29.5) | 26.2 (20.3–32.0) | 22.8 (17.1–28.5) | 24.6 (20.4–28.8) | 20.7 (16.0–25.5) | 22.2 (15.9–28.4) | 0.125 |
| Low/very low | 24.2 (19.3–29.1) | 18.8 (13.3–24.2) | 22.0 (16.5–24.2) | 20.4 (16.5–24.2) | 21.0 (16.4–25.7) | 21.8 (19.2–24.4) | 0.770 |
| SNAP participation |  |  |  |  |  |  |  |
| Participant | 15.7 (7.1–24.2) | 22.5 (18.1–27.0) | 16.9 (10.2–23.6) | 22.7 (18.6–26.9) | 20.8 (16.6–25.1) | 22.3 (18.2–26.4) | 0.338 |
| Eligible non-participant | 23.0 (19.6–26.3) | 23.6 (19.8–27.4) | 22.0 (17.4–26.6) | 23.1 (19.6–26.6) | 23.4 (18.1–28.7) | 20.8 (17.7–23.9) | 0.186 |
| Ineligible | 20.6 (18.1–23.1) | 20.6 (18.4–22.8) | 20.2 (18.5–22.0) | 21.1 (18.4–23.9) | 19.5 (17.1–21.9) | 21.9 (19.5–24.3) | 0.585 |

© 2017 by the authors. Submitted for possible open access publication under the
terms and conditions of the Creative Commons Attribution (CC BY) license (http://creativecommons.org/licenses/by/4.0/).
